# Rupture of non-communicating rudimentary horn at 35 weeks ending with a live birth: A case report

**DOI:** 10.1016/j.ijscr.2024.109641

**Published:** 2024-04-23

**Authors:** Wessam Taifour, Ghina Aljammal, Rafat Bhsass, Rasha Almnashef, Yousef Alshikh, Dema Adwan

**Affiliations:** a Faculty of Medicine, Damascus University, Damascus, Syria; b Obstetrics and Gynecology Hospital, Damascus University, Damascus, Syria

**Keywords:** Ruptured rudimentary horn pregnancy, Non-communicating horn, Unicornuate uterus, Mullerian duct, case report

## Abstract

**Introduction and importance:**

the rudimentary horn pregnancy frequently ruptured in the second and third trimesters during the normal process of rudimentary horn pregnancy (RHP) which results in a hemoperitoneum that may be fatal, however in very rare cases and under close observation, the pregnancy may continue and end with a live birth.

**Case presentation:**

A 30 years-old woman gravida 3, para 4 with no symptoms presented to the hospital at 30 weeks gestation for a routine examination and misdiagnosed as an abdominal ectopic pregnancy. The pregnancy continued for approximately 35 weeks, when a ruptured rudimentary horn pregnancy was discovered accidently during an emergency surgery, the left fallopian tube and ovary and the ruptured rudimentary horn were removed.

**Clinical discussion:**

Unicornuate uterus is a result of abnormal or failed development of one of the paired müllerian ducts, Spontaneous abortion in women with rudimentary horn pregnancy may occurs in the first and second trimester. Rudimentary horn pregnancies are associated with high maternal morbidity and mortality, And because of the risk of life-threatening complications, early diagnosis before rupture is essential for the successful management and prevention of these complications.

**Conclusion:**

The diagnosis of most cases of rudimentary horn pregnancy is considered challenging, and could be diagnosed after rupture, during emergency surgery. Although rudimentary horn pregnancy mostly ends in the first and second trimester, in some cases it may continue until term and end with a live healthy child.

## Highlights

• Rudimentary horn pregnancy rupture is an emergency case, and could end with death.
• Rudimentary horn pregnancy could misdiagnosed especially when unawareness of uterine malformations.
• Rudimentary horn pregnancy may continue until term and end with a live healthy child.

## 1 Introduction

The incomplete development of one Mullerian duct and the partial fusion of the contralateral side result in a Mullerian duct malformation called unicornuate uterus with a rudimentary horn [1]. Non-communicating rudimentary horn is a rare form of Mullerian duct malformation [2]. 40 % to 50 % of RHP cases had no symptoms, and only 8 % had been correctly diagnosed before symptoms appeared [3]. Continuation of pregnancy until the last trimester is very rare in this case, with high fetal and maternal death [4]. Here we report a case of a ruptured rudimentary horn pregnancy at 35 gestational age ending with live fetus, which was misdiagnosed as an abdominal ectopic pregnancy at 30 weeks and the patient was not aware of having uterine malformation, although she underwent two previous cesarean sections. Our work has been reported in line with the SCARE criteria [13].

## 2 Case presentation

**Fig. 1:**
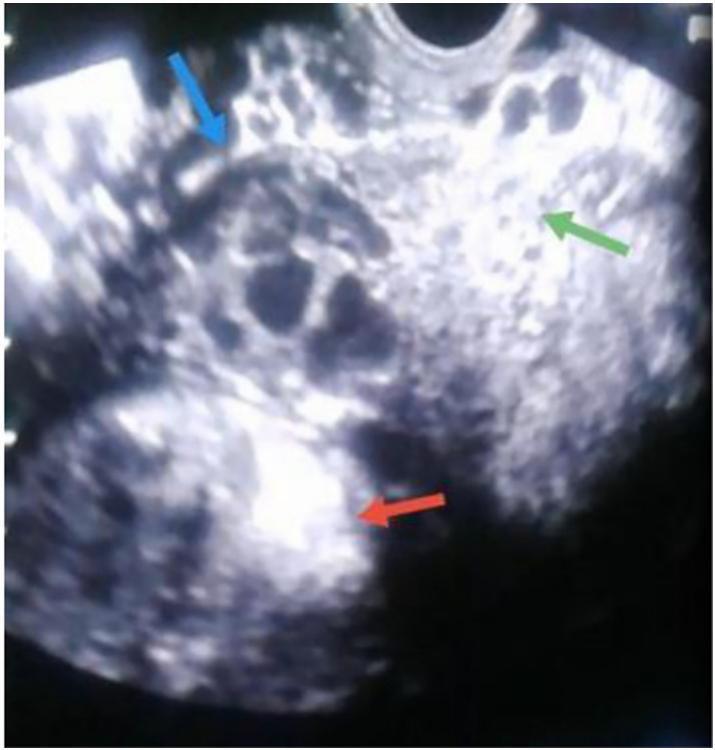
Transvaginal ultrasound (a retrospective diagnosis). Uterus (green arrow), rudimentury horn (blue arrow), placenta (red arrow). (For interpretation of the references to colour in this figure legend, the reader is referred to the web version of this article.)

**Fig. 2:**
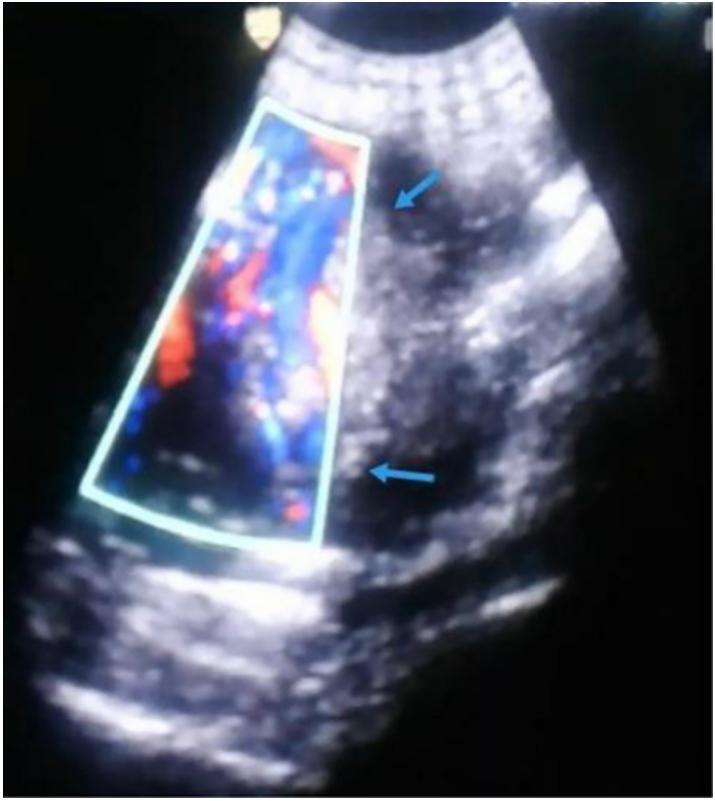
Doppler ultrasound shows placenta (blue arrows) with abnormal vascularization (increased and turbulent flow). (For interpretation of the references to colour in this figure legend, the reader is referred to the web version of this article.)

**Fig. 3:**
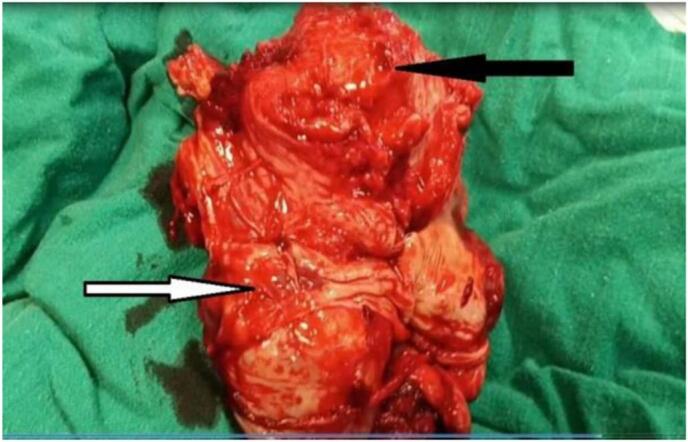
Placental tissue (black arrow) attached to the ruptured non-communicating rudimentary horn (white arrow).

A 30-year-old woman Gravida 4, Para 3 presented to the gynecology and obstetrics department at 30 weeks gestation for a routine examination. The patient was in good general health with no medical or family history, had no symptoms and had previously undergone two cesarean sections without complications, the last surgery was three years ago. The ultrasound examination had not been performed in the first and second trimester. On examination, there was no abdominal pain and the vital signs were stable. The ultrasound showed an empty uterus cavity with 3 cm endometrial thickness with live 30 weeks fetus positioning in abdomen with a small amount of fluid around it, fetal heartbeat was normal: 130 beats per minute, placental tissue outside uterus (Fig. 1) and the vascularization of the placenta was abnormal in Doppler ultrasound (increased and turbulent flow) (Fig. 2). The diagnosis of abdominal ectopic pregnancy was documented; however its location and connection to the viscera were not accurately detected. Magnetic resonance imaging (MRI) could not be done; the patient could not afford doing it due its high cost. The Laboratory results of blood hematological and renal profile analyses were within the normal range. The decision to do an elective cesarean section was made while the patient was under close observation; the patient had been given Dexamethasone 6 mg every 12 h to hasten the fetal lungs maturation. After 5 weeks of observation, an emergency laparotomy was decided due to a severe abdominal pain the patient was experiencing. Before the surgery, the blood hemoglobin level was 9.7 ml/dl, two blood units were transferred, the fetus was extracted from the abdomen, and a healthy newborn with normal Apgar sore was examined by pediatrician. The placenta was attached to the endometrium and muscular layer of the left rudimentary horn (Fig. 3). Due to the rupturing of the rudimentary uterine horn, the hemorrhage could not be controlled until the left fallopian tube and ovary were removed with the rupturing of the rudimentary uterine horn and placenta. The hemorrhage was controlled and patient was put under close observation for 24 h. After the surgery, the hemoglobin level was 10.7 ml/dl. The final diagnosis of the condition was positioned as a pregnancy in the left rudimentary uterine horn and the horn was ruptured during pregnancy. Pathology was consistent with non-communicating rudimentary horn pregnancy with evidence of placenta increta. The patient discharged from the hospital after 7 days in good health with her baby.

## 3 Discussion

The unicornuate uterus is a result of abnormal or failed development of one of the paired müllerian ducts. This group of anomalies can be further subdivided into 4 variants according to the American Fertility Society [8]. The isolated unicornuate uterus is the most common, with a reported frequency of 35 %, in case of rudimentary horn, it is noncavitary in 33 % of cases, cavitary noncommunicating in 22 % (like in our case), and cavitary communicating in 10 % [[1], [2], [3], [4], [5], [6], [7], [8], [9]]. Usually uterine abnormality has been linked to other defects like musculoskeletal defects, missing gallbladder and Hirschsprung disease [5], but no abnormality was reported in our patient. Typically, patients with Müllerian abnormalities present with pelvic pain, recurring pain, endometriosis, hematometra, and hematocolpos pregnancy loss [6]. Whereas, in our case, the patient was asymptomatic, came to the hospital for routine examination and she was not aware of having uterine malformation. Rudimentary horn pregnancy (RHP) is a rare case, occurs in approximately 1/76,000 to 1/150,000 pregnancies due to transperitoneal migration of sperm from the contralateral fallopian tube [10]. However, 40 % of women with RHP exhibit pregnancy related issues, such as first-and second trimester's abortion, preterm births, extra uterine pregnancy and intrauterine fetal death, a pregnancy can develop in both primitive noncommunicative and communicating horns [7]. Spontaneous abortion occurs in 24.3 % of women with rudimentary horn pregnancy in the first trimester and 9.7 % in the second trimester [8], in our case; the pregnancy proceeded for nearly 35 weeks before giving birth to a live baby. In addition, the fetus was positioned outside the cornu of the rupture non-communicating left rudimentary horn with the small amount of fluid around. A similar case to our study was reported in 2009, but the rudimentary horn rupture was discovered at 22 weeks gestational age [4]. There was another case where the pregnancy continued until 41 weeks and 3 days with fetus inside rudimentary horn without rupture [6].

Pregnancy outcomes are commonly classified as abortion, preterm delivery, uterine rupture and, rarely, full-term pregnancy [7]. Rudimentary horn pregnancies are associated with high maternal morbidity and mortality, with 50 % risk of uterine rupture which can lead to huge hemoperitoneum and could end with death [10], so because of the risk of life-threatening complications, early diagnosis before rupture is essential for the successful management and prevention of these complications. In the past, it was difficult to diagnose a rudimentary horn pregnancy and the diagnosis mostly was made accidentally in the operating room during surgery, when the gestational horn ruptures. Ultrasound could help in the diagnosis but its sensitivity alone has been noted to be low while MRI is a better diagnostic tool as it gives a good differentiation of the structures inside and outside the uterus and it can diagnose uterine anomaly [11], but in our case the patient was incapable of affording the high cost of MRI in a private center. Although patient had history of two cesarean sections but had no medical record and did not aware of her uterus abnormality. The main management is surgery with excision of the rudimentary horn and ipsilateral fallopian tube, some used methotrexate and intra cardiac potassium chloride preoperatively at early stages of pregnancy to reduce the size of pregnancy before interval excision of the rudimentary horn 6 weeks later [12].

## 4 Conclusion

The diagnosis of most cases of rudimentary horn pregnancy is considered challenging, and is diagnosed after rupture during emergency surgery, needing blood transfusion with increased morbidity and could end with death.

The reasons for misdiagnosis include a low index of suspicion or inappropriate skill at radiologic diagnosis, presentation in later gestation, or even unawareness of uterine malformations so we should keep in mind all the possibilities that are even remote and rare.

Although rudimentary horn pregnancy mostly ends in the first and second trimester, in some cases it may continue until term and end with a live healthy child.

It is the duty of the medical staff to inform the patient when they encounter any uterus deformation, and pregnant women should not hesitate to monitor her pregnancy regularly by echo.

## Consent

Written informed consent was obtained from the patient for publication of this case report and accompanying images. A copy of the written consent is available for review by the Editor-in-Chief of this journal on request.

## Ethical approval

The study is exempt from ethnical approval in our institution.

## Funding

This research did not receive any specific grant from funding agencies in the public, commercial, or not-for-profit sectors.

## Author contribution

**Rafat Bhsass**: study concept, data collection, writing the paper.

**Rasha Almnashef**: study concept, data collection, writing the paper.

**Ghina Aljammal**: study concept, data collection, writing the paper.

**Yousef Alshikh**: study concept, data collection, writing the paper.

**Wessam Taifour**: study conception and design.

**Dema Adwan**: study concept, Critical revision of the article.

All authors reviewed the results and approved the final version of the manuscript.

## Guarantor

Dema Adwan.

## Conflict of interest statement

We have no conflicts of interest to disclose.
